# Development and comparative evaluation of two antigen detection tests for Visceral Leishmaniasis

**DOI:** 10.1186/s12879-015-1125-3

**Published:** 2015-09-22

**Authors:** Aarthy C. Vallur, Yeung L. Tutterrow, Raodoh Mohamath, Sowmya Pattabhi, Asrat Hailu, Asim O. Abdoun, Abdalla E. Ahmed, Maowia Mukhtar, Md Abdus Salam, Meirielly Lima Almeida, Roque P. Almeida, Dinesh Mondal, Audrey Albertini, Hashim Ghalib, Malcolm S. Duthie, Steven G. Reed

**Affiliations:** Infectious Disease Research Institute, 1616 Eastlake Ave E, Seattle, WA 98102 USA; Department of Microbiology, Immunology and Parasitology, Addis Ababa University, Addis Ababa, Ethiopia; Institute of Endemic Diseases, University of Khartoum, Khartoum, Sudan; Department of Microbiology, Rajshahi Medical College, Rajshahi, Bangladesh; Department of Medicine, Universidade Federal de Sergipe, Aracaju, Sergipe Brazil; International Center for Diarrhoeal Diseases Research, Dhaka, Bangladesh; Foundation for Innovative New Diagnostics (FIND), Geneva, Switzerland

**Keywords:** Diagnosis, *Leishmania*, Antigen, Treatment, Antibody, Kala azar, Infection

## Abstract

**Background:**

Visceral leishmaniasis (VL) can be fatal without timely diagnosis and treatment. Treatment efficacies vary due to drug resistance, drug toxicity and co-morbidities. It is important to monitor treatment responsiveness to confirm cure and curtail relapse. Currently, microscopy of spleen, bone marrow or lymph node biopsies is the only definitive method to evaluate cure. A less invasive test for treatment success is a high priority for VL management.

**Methods:**

In this study, we describe the development of a capture ELISA based on detecting *Leishmania donovani* antigens in urine samples and comparison with the *Leishmania* Antigen ELISA, also developed for the same purpose. Both were developed as prototype kits and tested on patient urine samples from Sudan, Ethiopia, Bangladesh and Brazil, along with appropriate control samples from endemic and non-endemic regions. Sensitivity and specificity were assessed based on accurate detection of patients compared to control samples. One- Way ANOVA was used to assess the discrimination capacity of the tests and Cohen’s kappa was used to assess their correlation.

**Results:**

The *Leishmania* Antigen Detect™ ELISA demonstrated >90 % sensitivity on VL patient samples from Sudan, Bangladesh and Ethiopia and 88 % on samples from Brazil. The *Leishmania* Antigen ELISA was comparable in performance except for lower sensitivity on Sudanese samples. Both were highly specific. To confirm utility in monitoring treatment, urine samples were collected from VL patients at days 0, 30 and 180 post- treatment. For the *Leishmania* Antigen Detect™ ELISA, positivity was high at day 0 at 95 %, falling to 21 % at day 30. At day 180, all samples were negative, corresponding well with clinical cure. A similar trend was also seen for the *Leishmania* Antigen ELISA albeit; with lower positivity of 91 % at Day 0 and more patients, remaining positive at Days 30 and 180.

**Discussion:**

The *Leishmania* Antigen Detect™ and the *Leishmania* Antigen ELISAs are standardized, user- friendly, quantitative and direct tests to detect *Leishmania* during acute VL as well as to monitor parasite clearance during treatment. They are a clear improvement over existing options.

**Conclusion:**

The ELISAs provide a non-invasive method to detect parasite antigens during acute infection and monitor its clearance upon cure, filling an unmet need in VL management. Further refinement of the tests with more samples from endemic regions will define their utility in monitoring treatment.

## Background

Visceral Leishmaniasis (VL) is a sand fly-borne disease caused by infection with protozoan parasites of the *Leishmania donovani* complex. The vast majority of the 300,000 estimated annual cases are reported from focal regions in Ethiopia, Sudan, South Sudan, India, Bangladesh and Brazil, though the disease is also endemic in the Mediterranean basin [1]. VL incidence is highest in the Indian sub- continent, followed by East Africa, where the causative organism is *L. donovani* and transmission is anthroponotic. In Brazil and the Mediterranean basin, the disease is caused by *L. infantum*, while transmission is zoonotic, with dogs serving as an intermediary host [1]. VL incidence has also been reported in previously non- endemic regions owing to travel to and migration from endemic regions and environmental conditions that have expanded the habitat of the sand fly [2–4]. Current diagnosis of VL is based on presentation of clinical symptoms such as fever, splenomegaly and weight loss, then confirmed by parasite detection in bone marrow/splenic biopsy in Africa and South America; or detection of antibodies against the rK39 antigen in the Indian subcontinent [5].

Multiple drug regimens are available to treat VL. Antimoniates including sodium stibogluconate (SSG) and meglumine antimoniate (MEG) are the first line drugs in Brazil and Africa, while resistance to antimoniates have phased them out in the Indian sub- continent, where amphotericin B, paromomycin and miltefosine are the drugs of choice [6, 7]. Liposomal amphotericin B (AmBisome™) is now preferred in Europe and the Indian sub- continent [8].

Timely diagnosis and treatment are imperative as, without treatment, VL can be fatal [1, 6]. Drug regimens are expensive and can cause severe side effects [5, 6, 9]. These factors have contributed to low compliance in many VL endemic regions, leading to unresponsiveness and the emergence of drug- resistant parasite strains [7, 9]. It is important to monitor treatment and detect unresponsiveness as early as possible. Treatment failure ranges from 3–10 % in immune competent individuals and 50–60 % in immune compromised individuals [4, 10]. Though simple tests are available to accurately confirm VL disease, none of the current diagnostic tests is suitable to monitor treatment or cure [11]. Commonly used diagnostic tests such as DAT and the rK39 rapid detection test (RDT) cannot differentiate between past and current infections because the antibodies detected persist after clinical cure is achieved [12]. Microscopy of splenic or bone marrow biopsies, though confirmatory for parasite clearance is not a practical tool to monitor treatment due to the painful and invasive nature of sampling.

A non-invasive, standardized test for monitoring treatment success in a clinical setting would complement tools to confirm VL, and aid in effectively managing VL. Such a test should ideally detect parasite or parasite products as a measure of infection since presence of parasite products should theoretically correlate with parasite burden. Hence, a drop in antigen levels as measured by the test would reflect a decrease in parasite burden due to anti-*Leishmania* treatment and eventual clearance of parasites. It must also be sensitive, specific, easy to use, quantitative and preferably non- invasive for repeated sample collection.

At present, KAtex is the only commercially available *Leishmania* antigen detection test [13]. Although highly specific, KAtex’s sensitivity has been variable, limiting its widespread use for the assessment of treatment [14–17]. Knowing that *Leishmania* antigens are excreted in the urine of VL patients, we developed a sensitive urine-based test to detect antigens with which to evaluate treatment [18, 19]. We compared its performance to a similar product developed by Kalon Biological Ltd., UK. We discuss the validation of the antigen detection tests and their evaluation for determining VL cure post- treatment.

## Methods

### Samples

Urine samples of VL patients were collected as part of routine diagnosis and treatment. Except the post- treatment samples, all VL patient samples were collected at diagnosis prior to treatment start. Samples were collected at Gedaref Hospital, Sudan the Rajshahi Medical College Hospital, Bangladesh and at the clinic in Sergipe, Aracaju, Brazil. Urine samples from Ethiopia were collected in Southern Ethiopia during ongoing field studies. Each of the Ethics Committees of Khartoum University, Rajshahi Medical College, University of Sergipe and Addis Ababa University approved study protocols, respectively. Written or verbal informed consent was obtained from patients at the time of collection. Inclusion criteria for VL patients in Ethiopia, Sudan and Brazil were presentation of clinical symptoms and demonstration of parasites in spleen, bone marrow, or lymph node smear or positive rK39 for Bangladesh. Urine samples from patients with other diseases (OD) were kindly provided by FIND, Geneva and consisted of 10 each from patients with human African trypanosomiasis (HAT) and *P. falciparum* malaria from Uganda, and 10 from TB patients in Thailand. For all samples provided by FIND, written, informed consent was obtained at the time of collection. Non-endemic control (NEC) samples (*n* = 49) were collected from local volunteers in Seattle, USA or purchased from Equitech-Bio, Inc. (Kerrville, TX). Urine samples from 10 healthy endemic controls (EC) were also obtained from Bangladesh as evidenced by lack of symptoms and a negative rK39-RDT. All urine samples were stored frozen and transported on dry- ice to minimize any adverse effects of freeze- thawing.

### Generation of anti-*Leishmania* antibodies

*L. donovani* (MHOM/SD/00/1S-2D) promastigotes were seeded in culture flasks and cultured at 25 °C in M199 medium (Sigma, St. Louis, MO) supplemented with 10 % FCS (Hyclone), 1XM199 Hanks salt, 1XMEM amino acids Solution (Invitrogen), 10 mM MEM non-essential amino acids (Sigma), 40 mM HEPES pH 7.4, 0.1 mM adenine, 5 μg/mL hemin, 1.5 uM biotin, 100 U/mL penicillin, 100 μg/mL streptomycin, 2 mM L-glutamine, 0.35 mg/mL sodium biocarbonate at final pH 7.2 for 10 days. Stationary phase promastigotes at a density of 2-4×10^7^ parasites/mL were harvested and washed three times with cold PBS and frozen at −80 °C. To prepare lysates, the pelleted parasites were and resuspended in 10 mM Tris–HCl, 1 mM EDTA (pH 8.0) containing 1X Halt protease inhibitor (ThermoScientific) at 1×10^9^ parasites/mL. Whole cell lysate (WCL) was prepared by freeze thawing the pellet in liquid nitrogen (6X) followed by three rounds of sonication for 30 s at 10 Hz. Soluble lysate antigen (SLA) was obtained by further centrifuging at 15,000 rpm for 45 min and discarding the insoluble pellet.

To generate antibodies, New Zealand white rabbits (NZW) with low residual reactivity to WCL were selected and immunized with 0.5 mg WCL added complete Freund’s adjuvant (Sigma) followed by 3 booster immunizations with 0.25 mg WCL with incomplete Freund’s adjuvant (Sigma) at 3 week intervals. Blood was collected 2 weeks after the final boost (R & R Research, LLC., Stanwood, WA).

### Affinity purification and labeling

Total IgG was purified from the anti-sera of three rabbits with high IgG titers using Protein G Sepharose. Total IgG was further affinity purified against SLA coupled with Cyanogen bromide (CNBr)-activated-Sepharose™ 4B (GE Healthcare). Affinity purified antibodies were conjugated with horseradish peroxidase (HRP labeling kit, Thermofisher) at a 6:1 molar ratio using sodium periodate (NaIO_4_) and sodium cyanoborohydride (NaBH_4_CN) for oxidation and reduction, respectively. Protein concentration was determined using Bradford’s method and 0.77 volume of glycerol was added to the conjugated antibodies before storage at −20 °C.

### Capture ELISA optimization

ELISA conditions were optimized in the context of ELISA plate selection, capture and detection antibody concentrations, urine sample dilution as well as sample incubation duration. In brief, Immulon™ 2HB plates were coated with 1 μg/mL affinity purified antibodies in 0.1 M sodium bicarbonate- carbonate buffer (pH9.6) at 4 °C overnight. After blocking with 1 % BSA-PBS/PBS-T, 50 uL of 1:1 diluted urine samples were added and allowed to incubate at room temperature for 1 h on a shaker. After 5 washes with 1X PBS/PBS-T, 100 μL of HRP-labeled anti-SLA IgG was added to each well at 1:1000 dilution and the plate was incubated at room temperature for 1 h. After five washes, 100 μL of SureBlue™TMB peroxidase substrate (KPL, Inc.) was added and incubated for 5 min before the reaction was stopped with 50 μL of 1 N H_2_SO_4_. Optical density was read at 450 nm (VersaMax microplate reader, Molecular devices) immediately. Affinity purified and labeled antibodies were then transferred to InBios International Inc., Seattle for developing the Leish Antigen Detect™ ELISA.

### *Leishmania* Antigen Detect™ ELISA

ELISA was performed according to the manufacturer’s instructions (InBios International Inc. Seattle, WA). In brief, plate controls (positive and negative) and test samples were diluted with ELISA dilution buffer at 1:1 ratio and 50 μl added to duplicate wells for incubation at 37 °C for 30 min. After 6 washes, 50 μl of HRP-*Leishmania* ELISA conjugate was added to each well and allowed to incubate at 37 °C for 30 min. After washing, 75 μl of TMB solution was added to each well for 10 min. Reactions were terminated by adding 50 μl of stopping solution, and plates were read immediately at 450 nm. Each plate was assessed passed based on the discrimination between the signals observed for the in-built positive and negative controls, with criterion being a Positive control/ negative control >5. For determining the specificity of the ELISA, ROC curves were generated as described below. For determining positivity of samples, cut- offs were generated based on a panel of NEC samples run in each plate. Samples with signals over the cut- off were deemed positive.

### KAtex

KAtex agglutination tests were conducted following manufacturer’s instructions (Kalon Biological Ltd., Guildford, UK), with minor modifications. In brief, 200 μl of freshly thawed urine was transferred into a collection tube, submerged in boiling water for five minutes and then cooled to room temperature. 50 μl of the boiled urine was added to the reaction zone on the glass slide and mixed with one drop of well-mixed latex. The liquids were mixed with a toothpick and spread to cover the entire surface of the reaction zone. The glass slide was tilted with a rotating action, and the degree of agglutination interpreted as instructed.

### *Leishmania* Antigen ELISA

*Leishmania* antigen ELISA produced by Kalon Biologicals Ltd., UK was provided by FIND, Geneva, Switzerland. Polyclonal antibodies were prepared from antiserum of sheep immunized with 4×10^9^ cultured promastigotes from *Leishmania donovani* strains from Sudan (LV9), Nepal (BPK282). Antisera were purified using an antigen affinity column prepared using concentrated BPK 282 spent cell culture. Kits were used according to the manufacturer’s instructions. Briefly, samples were diluted 1:20 in assay diluent, after which 100 ul/well of these and antigen calibrators were added to 96-well plates in triplicate or duplicate, respectively. Plates was incubated for 30 min at 37 °C, then washed 5 times before the addition of 100 uL of 1X Tracer. After 30 min of incubation at 37 °C plates were washed and 100 ul/well TMB Substrate solution was added for 30 min. Reactions were terminated by adding 100 ul/well of Stop solution. Plates were then read immediately at 450 and 620 nm (VersaMax microplate reader, Molecular Devices). Resulting OD was obtained by subtraction of OD at 620 nm from OD at 450 nm. Urinary antigen unit (UAU)/mL of samples were extrapolated from a four parameter logistic standard curve constructed in Excel using the mean values obtained for the calibrators. Samples with UAU less than the lowest calibrator (2 UAU/mL) were considered negative.

### Calculations and statistical analysis

GraphPad Prism six software was used for generating Receiver-operating characteristic (ROC) curves and statistical analysis. ROC curves were generated with non-VL (NEC, OD) and VL samples to determine thresholds that afforded the best specificity. Sensitivity was calculated as the percentage of VL samples correctly assayed as positive while specificity was calculated as the percentage of non-VL samples (NEC, OD) correctly assayed as negative. Area under the curve (AUC) used to assess the accuracy of each test. One-way ANOVA (Dunnett’s multiple comparison) was performed between VL and non- VL samples with *p* values ≤0.05 were considered statistically significant. Cohen’s kappa values were used to determine the correlation between the ELISAs.

## Results

### Capture ELISA validation

**Fig. 1:**
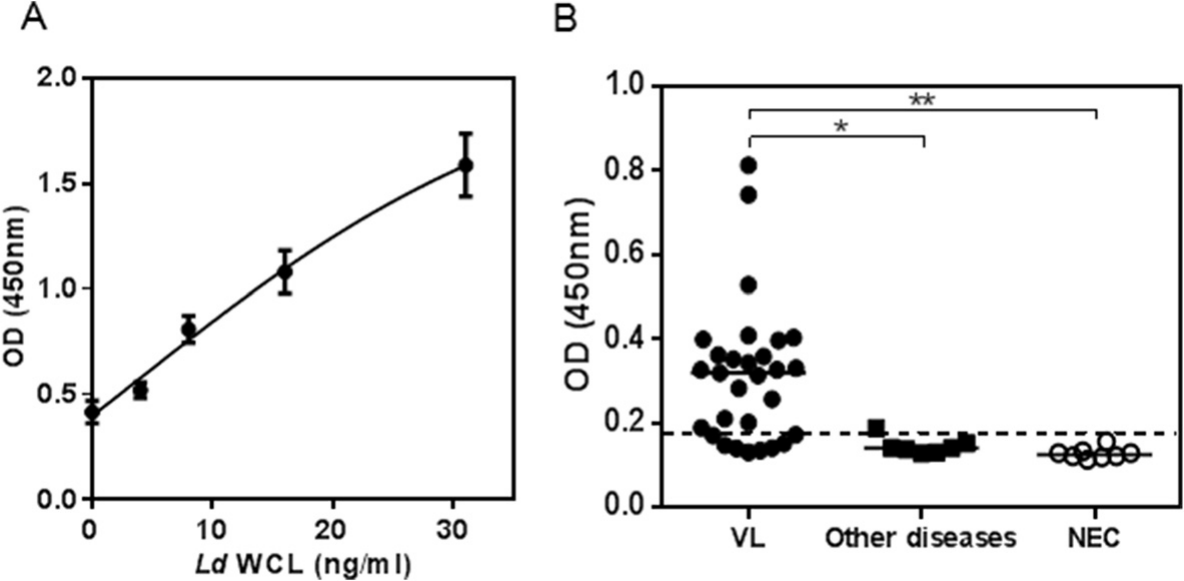
Optimization of antigen capture ELISA using affinity purified and labeled antibody pair. **a**. Standard curve was generated using pooled urine from NEC spiked with 0, 4, 8, 16 and 32 ng/mL of *Ld* WCL for determining limits of detection of the antibody pair. **b**. Performance of capture ELISA on urine samples from VL patients (*n* = 29), OD (*n* = 7) and NEC (*n* = 8). * and ** represent significant p-values < 0.05, as calculated by One-way ANOVA

To establish the performance of the affinity purified antibody pair for incorporation into a capture ELISA test, initial assays using urine samples either spiked with WCL or from confirmed VL patients were used. A serial dilution of WCL added to pooled urine samples from healthy non- endemic controls (NEC) ranging from 4–32 ng/mL was used to estimate the detection limits (Fig. 1a). Robust signals were observed across the concentration range with the lower limit of detection being 4 ng/mL. Hence a broad range of detection was obtained with the antibody pair.

We then tested whether *Leishmania* antigens could be detected in the urine of VL patients. Compared to urine from healthy NEC, samples from 29 confirmed VL patients (12 from Sudan, six from Bangladesh and 11 from Brazil) had significantly higher signals, with 22/29 (76 %) testing positive (Fig. 1b). Detection of VL patients was also highly specific, with signals being significantly higher than those of patients with other diseases (OD). Taken together, these data indicated that, the antibody pair could specifically detect *Leishmania* antigens in urine in a laboratory-based capture ELISA.

### Performance of *Leishmania* Antigen Detect™ ELISA

**Fig. 2:**
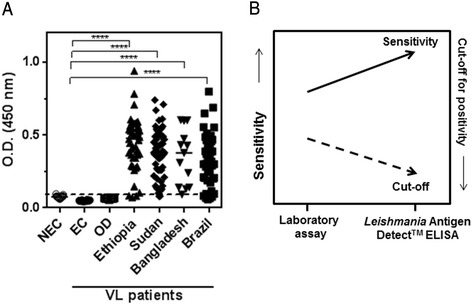
Performance of *Leishmania* Antigen Detect™ ELISA. **a**. Performance of *Leishmania* Antigen Detect™ ELISA on urine samples from VL patients in Ethiopia (*n* = 46), Sudan (*n* = 64), Bangladesh (*n* = 13) and Brazil (*n* = 43) compared to NEC (*n* = 49), EC from Bangladesh (*n* = 10) and OD (*n* = 30). OD consists of samples from patients with HAT (*n* = 10), *P. falciparum* malaria (*n* = 10) and tuberculosis (*n* = 10). Lines represent median. Dotted line indicates cut-off value for positivity as calculated from the mean of NEC added three standard deviations. **** represents significant p-values < 0.01, as calculated by One-way ANOVA. **b**. The standardized *Leishmania* Antigen Detect™ ELISA offers higher sensitivity for VL patient samples and lower background reactivity when compared to the same reagents used in a laboratory- based assay

The antibody pair characterized in a laboratory-based capture ELISA was then incorporated into ready-to-use, standardized *Leishmania* Antigen Detect™ ELISA kits with in- built positive and negative controls, optimized reagents and assay steps. We assessed the performance of the *Leishmania* Antigen Detect™ ELISA on an expanded panel of urine samples from VL patients in 4 major endemic regions although only 13 urine samples were available from the Indian subcontinent compared to 46, 64 and 43 from Ethiopia, Sudan and Brazil, respectively. *Leishmania* antigens were readily detected in the majority of urine samples from VL patients from Ethiopia, Sudan and Bangladesh where *L. donovani* is the causative agent, with significantly higher signals than NEC and OD (Fig. 2). Responses were also significantly higher than the 48 NEC, 10 EC and 30 OD samples (Fig. 2). Based on the cut- off for positivity generated against the non- VL samples, high sensitivities of 100 % for Bangladesh (95 % CI of 75.3-100), 96.9 % for Sudan (95 % CI of 89.2-99.6), 93.5 % for Ethiopia (95 % CI of 82.1-98.6) and 88.4 % for Brazil (95 % CI of 77.8-96) were calculated for the *Leishmania* Antigen Detect™ ELISA (Table 1). Considering the control samples evaluated, the specificity of the assay was 100 % (95 % CI of 90.3-100 for NEC and 69.2-100 for OD) (Table 2).

**Table 1:** Sensitivity of the *Leishmania* Antigen Detect™ ELISA and the Leishmania Antigen ELISA on VL patient urine compared to KAtex

	Leishmania Antigen™ Detect ELISA Sensitivity % (95% CI)	Leishmania Antigen ELISA Sensitivity % (95% CI)	KAtex %
Ethiopia (*n*=46)	93.5 (82.1-98.6)	87.0 (73.7-95.1)	61 (45.4-74.9)
Sudan (*n*=64)	96.9 (89.2-99.6)	78.1 (66.0-87.5)	63 (49.5-74.3)
Bangladesh (*n*=13)	100 (75.3-100)	76.9 (46.2-95.0)	69 (38.6-90.7)
Brazil (*n*=43)	88.4 (77.8-96)	81.4 (66.6-91.6)	56 (39.9-70.9)

**Table 2:** Specificity of the *Leishmania* Antigen Detect™ ELISA and *Leishmania* Antigen ELISA

Samples	Leishmania Antigen Detect™ ELISA Specificity % (95% Cl)	Leishmania Antigen ELISA Specificity % (95% Cl)
NEC (*n*=36)	100 (90.3–100)	100 (90.3–100)
EC (*n*=10)	100 (69.1–100)	90 (55.5–99.7)
HAT (*n*=10)	100 (69.2–100)	100 (69.2–100)
Pf Malaria (*n*=10)	100 (69.2–100)	70.0 (34.8–93.3)
TB (*n*=10)	100 (69.2–100)	80.0 (44.4–97.5)

We observed that the sensitivity of the test was enhanced when transferred form a laboratory-based capture ELISA assay (76 %) to the standardized *Leishmania* Antigen Detect™ ELISA (>90 %) which was largely due to the increased discrimination between controls and VL patients (Fig. 1b and Table 1). This is borne out by the lower background reactivities to the NEC and OD samples and the two-fold lower cut- off value for positivity, observed with the standardized *Leishmania* Antigen Detect™ ELISA (0.12) compared to the laboratory developed capture ELISA (0.25) (Figs. 1b and 2a). The enhanced performance further attests to the value of standardized kits in diagnostics over laboratory- based assays (Fig. 2b). Altogether, this data demonstrate that the *Leishmania* Antigen Detect™ ELISA can detect VL specific antigens in urine samples from the major endemic regions.

### Comparable performance of the *Leishmania* Antigen ELISA

**Fig. 3:**
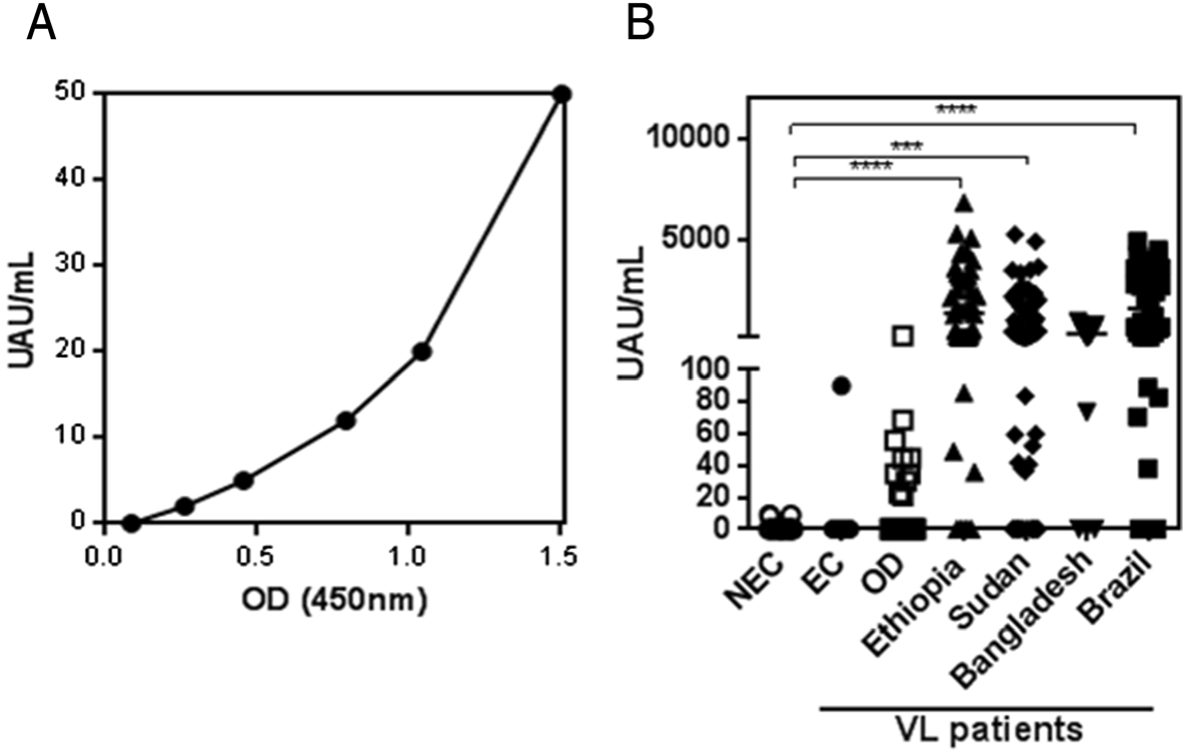
Performance of *Leishmania* Antigen ELISA. **a**. Standard curve representing means of the signals obtained in the Leishmania Antigen ELISA using the calibrated standards provided with the kit. **b**. Performance of *Leishmania* Antigen ELISA on urine samples from VL patients in Ethiopia (*n* = 46), Sudan (*n* = 64), Bangladesh (*n* = 13) and Brazil (*n* = 43) compared to NEC (*n* = 49), EC from Bangladesh (*n* = 10) and OD (*n* = 30). OD consists of samples from patients with HAT (*n* = 10), *P. falciparum* malaria (*n* = 10) and tuberculosis (*n* = 10) in UAU/mL. Lines represent median

To provide a sensitive and quantitative assay, Kalon Biological, Ltd., UK generated a prototype antigen-detection capture ELISA using sheep anti-*Leishmania* antibodies. In the *Leishmania* Antigen ELISA, antigen concentrations are calibrated from a standard curve with known concentration of antigens and expressed as Urine Antigen Units (UAU)/mL (Fig. 3a). Measured UAU/mL in urine samples from VL patients were significantly higher in Ethiopia, Sudan and Brazil compared to NEC, EC and OD (Fig. 3b). Sensitivities of 77 % for Bangladesh (95 % CI of 46.2-95.0), 78.1 % for Sudan (95 % CI of 66.0-87.5), 87 % for Ethiopia (95 % CI of 73.7-95.1) and 81 % for Brazil (95 % CI of 66.6-91.6) were calculated for the *Leishmania* Antigen ELISA (Table 1). However, two urine samples from patients with OD, notably TB and malaria were positive by the *Leishmania* Antigen ELISA, displaying antigens well above the lower threshold of detection defined by the standard curve while three other malaria samples were borderline positive, harbouring between 40-45UAU/mL (Fig. 3b). Similarly, one EC sample from Bangladesh was also positive. Hence the specificity of the ELISA was100% on NEC and HAT samples, while varied from 70–90 % on the TB, *Pf* malaria and EC samples used, (Table 2). The agreement between the tests also suggested a slightly higher sensitivity of the *Leishmania* Antigen Detect™ ELISA (Table 3). Cohen’s kappa revealed good correlation between the tests on samples from Ethiopia and moderate correlation on samples from Brazil (Table 3). Overall, the performance of the *Leishmania* antigen ELISA from Kalon was comparable to the *Leishmania* Antigen Detect™ ELISA on VL patients.

**Table 3:** Agreement between *Leishmania* Antigen Detect™ ELISA and the *Leishmania* Antigen ELISA on VL patient urine samples from Ethiopia, Sudan, Brazil and Bangladesh are tabulated

Test Agreement		Leishmania Antigen Detect™ ELISA		Correlationf/c) 95% Cl
		Positive	Negative	
Ethiopia (*n*=46)				
Leishmania Antigen ELISA	Positive	35	0	0.802 (0.59- 1.00)
	Negative	3	8	
Sudan (*n*=64)				
Leishmania Antigen ELISA	Positive	50	0	0.207 (-0.04-0.45)
	Negative	12	2	
Brazil (*n*=43)				
Leishmania Antigen ELISA	Positive	34	1	0.551 (-0.21-0.89)
	Negative	4	4	
Bangladesh (*n*=13)				
Leishmania Antigen ELISA	Positive	10	0	Nd
	Negative	3	0	

### Performance of ELISAs compared to KAtex

KAtex is the only commercially available antigen detection test and is based on detecting a *Leishmania-*specific carbohydrate in urine [13]. To provide a direct comparison in assay performance the expanded urine panel used for the two ELISA kits was also evaluated with KAtex. While KAtex also had a high specificity of 92 % for VL samples, only moderate sensitivities at 61 % for Ethiopia (95 % CI of 45.4-74.9), 63 % for Sudan (95 % CI of 49.5-74.3), 69 % for Bangladesh (95 % CI of 38.6-90.7) and 56 % for Brazil (95 % CI of 39.9-70.9) were obtained (Table 1). The *Leishmania* Antigen Detect™ ELISA and *Leishmania* Antigen ELISA from Kalon were more sensitive for VL samples than KAtex.

### Utility of antigen detection tests in monitoring VL treatment success

**Fig. 4:**
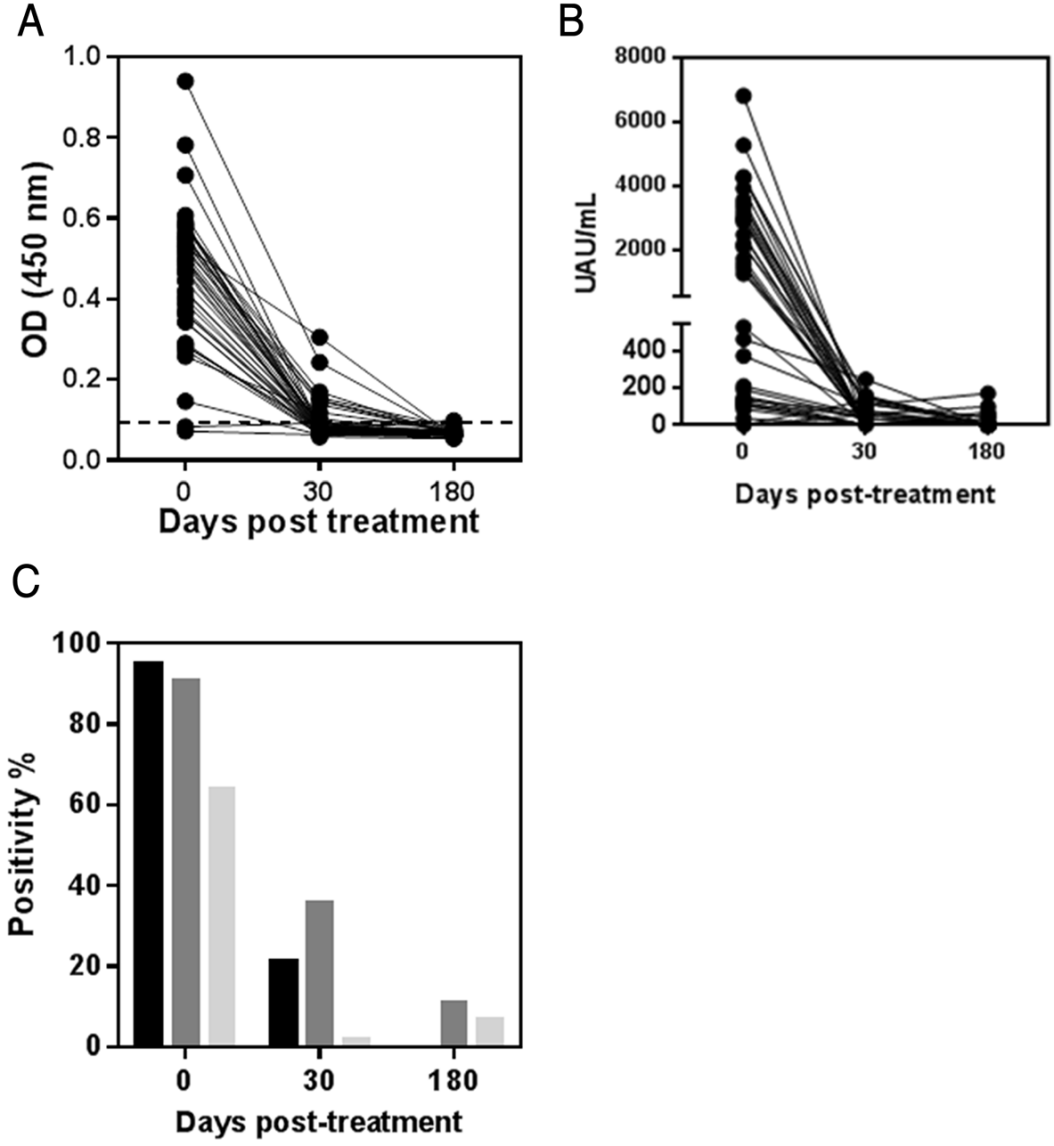
Performance of *Leishmania* Antigen Detect™ ELISA and *Leishmania* Antigen ELISA on treatment follow- up urine samples from Ethiopia. **a**. Mean ELISA signals of 42 VL patients undergoing SSG, MEG or L-AmB treatment in Ethiopia at initiation (Day 0) and post initiation (Days 30 and 180) of treatment as measured by the *Leishmania* Antigen Detect™ ELISA. Dotted line indicates cut-off value for positivity as calculated from the mean of negative controls added 3 standard deviations. **b**. UAU/mL for 42 VL patients undergoing SSG, MEG or L-AmB treatment in Ethiopia at initiation (Day 0) and post initiation (Days 30 and 180) of treatment as measured by the *Leishmania* Antigen ELISA **c**. Positivity percentage of the *Leishmania* Antigen Detect™ ELISA (Black bars) and *Leishmania* Antigen ELISA (dark gray bars) compared to KAtex (light gray bars) on VL patient urine samples from Ethiopia at initiation (Day 0) and post initiation (Days 30 and 180) of treatment

To assess the potential of the *Leishmania* Antigen Detect™ ELISA and the *Leishmania* antigen ELISA from Kalon for monitoring treatment, we evaluated a set of urine samples from 42 confirmed Ethiopian VL patients starting at diagnosis prior to treatment start (Day 0) through Day 30 and up to Day 180 post-treatment. Patients were provided a standard 28 day treatment course of SSG, with the exception of two who were treated with AmBisome™ and one with MEG. Initial cure and clinical cure were evaluated at 30 and 180 days, respectively, by resolution of clinical symptoms and absence of parasites by microscopy. At Day 0, 40/42 patients were positive with a median response of 0.5 (Fig. 4a). The 2 patients who were negative by the *Leishmania* Antigen Detect™ ELISA at Day 0 had low parasite grade (1+ and 2+) by microscopy (data not shown). At Day 30, there was a significant drop in the median to 0.1 and a concomitant drop in positivity to 9/42 patients (Fig. 4a). The 9 patients who were still positive at Day 30 had significantly higher parasite grades at diagnosis (4+ to 5+). None of the patients tested positive at Day 180 (Fig. 4a). The decline in response thus, reflected parasite clearance and clinical cure.

The samples were also evaluated with *Leishmania* Antigen ELISA and KAtex. The trend of UAU/mL was very similar to the signals observed with the *Leishmania* Antigen Detect™ ELISA, though the sensitivity at Day 0 was slightly lower. At Day 0, 38/42 patients tested positive with a median value of 1,472 UAU/mL which rapidly decreased by Day 30, when only 15/42 patients were still positive with significantly less urinary antigens, ranging from 33–248 UAU/mL (Fig. 4b). By Day 180, except for five patients, all were negative with no detectable urine antigens (Fig. 4b). The antigens detected in the positive patients ranged from 40–172 UAU/mL with one being borderline positive, with 40–45 UAU/mL. KAtex was less sensitive than ELISAs for diagnosis of confirmed VL patients at Day 0, when sensitivity was >90 % for both ELISAs compared to only 64 % by KAtex (Fig. 4c). At Day 30, only 2 % were positive by KAtex compared to 21 % by the *Leishmania* Antigen Detect™ ELISA and 36 % by the *Leishmania* Antigen ELISA (Fig. 4c). At Day 180, 7 % of the patients were still positive by KAtex and 11 % by the *Leishmania* Antigen ELISA but none by the *Leishmania* Antigen Detect™ ELISA (Fig. 4c). Based on this data set, the *Leishmania* Antigen Detect™ ELISA and the *Leishmania* Antigen ELISA were more sensitive than KAtex for initial detection of active VL and appear to be better tools for monitoring treatment efficacy.

## Discussion

Early diagnosis and efficacious treatment are the keys to VL management. VL treatment options are plagued by high costs, severe systemic side effects and unresponsiveness. Though simple and accurate diagnostic tests are widely available to confirm VL, they are not suitable to monitor treatment efficacy and cure. According to the WHO’s recommendations, tests for treatment efficacy and cure should be the “highest research priority” in VL control [20]. At this time, microscopy and KAtex are the only available tests to follow treatment [11]. Though attempts have been made to develop capture ELISAs to detect antigens in the urine of VL patients, they have not yet progressed to standardized tests [21].

With the goal of developing a *Leishmania* antigen detection test suitable for following treatment, we developed the *Leishmania* Antigen Detect™ ELISA. As a tool for monitoring treatment, the *Leishmania* Antigen Detect™ ELISA was sensitive, specific and suggested parasite clearance during clinical cure (Fig. 3). Thus, in terms of performance, the test represents a significant improvement over current options. The ELISA also has practical advantages over KAtex such as not requiring the boiling of urine, thus improving convenience of the assay. The profile of *Leishmania* antigens in the urine of VL patients can be complex with not one dominating antigen detectable at all times [18]. The *Leishmania* Antigen Detect™ ELISA contains antibodies raised against a diverse panel of antigens derived from *L. donovani*. The diversity of the antibody repertoire could be a reason for the high sensitivity of the *Leishmania* Antigen Detect™ ELISA compared to KAtex and other previous capture ELISAs, both in- house and documented, which were developed to detect single antigens [21, 22] (data not shown). The *Leishmania* Antigen Detect™ ELISA also performed comparably to the *Leishmania* Antigen ELISA, an antigen detection ELISA developed by Kalon Biological Ltd. as an improvement over their KAtex test. The Katex and Kalon VL ELISA are both based on polyclonal antibody response to whole promastigotes. However the *Leishmania* Antigen ELISA utilizes affinity purified polyclonal antibodies based on an antigen similar to the whole cell lysate. The format for both ELISAs is similar, and so are their performances on urine samples from VL patients and controls (Table 1). However, the *Leishmania* Antigen Detect™ ELISA was more specific for VL based on the OD samples tested in this study (although endemic controls were not tested) as well as more sensitive on VL samples from endemic regions in which *L. donovani* is the causative agent, namely Sudan and Bangladesh.

Early detection of treatment non- responsiveness could help alter treatment options in time to prevent adverse outcomes such as mortality. It may be possible to refine and use the ELISAs as a means of identifying treatment non- responsiveness, if detection of antigen in urine can be considered a surrogate for parasite burden. A concern for VL patients infected with *L. donovani* is the potential of developing PKDL after clinical cure [23, 24]. As much as 50 % of treated VL patients in Sudan, South Sudan and Northern Ethiopia develop PKDL, 6 months after treatment [24]. It is probable that antigen excretion in urine reflects parasite clearance by the drug, which occurs within 26 days for both antimoniates and Amphotericin B [25–28]. None of the treated patients in this study relapsed or developed PKDL as of this time. Since these patients were from Southern Ethiopia where relapse and PKDL are rare compared to other regions of East Africa, it is not possible to draw any conclusions regarding the dynamics of antigen presence in urine and the risk for PKDL development. The testing of PKDL and Cutaneous Leishmaniasis urines would be useful to determine if both these two ELISAs are specific to Visceral Leishmaniasis.

Though originally developed as a tool to monitor treatment success, the high sensitivity of the ELISAs on VL patient samples from diverse endemic regions indicates that it also has potential as a tool for primary diagnosis. It could serve as an alternative in cases where currently used diagnostic tests are not as effective. Such a situation can arise in individuals with low antibody titers to diagnostic antigens like rK39, and immune suppression co- morbidities such as HIV [14, 29]. Accurate diagnosis in such difficult scenarios can significantly aid case management. The *Leishmania* Antigen ELISA (Kalon Biological Ltd.) was designed as a proof of cure and test for treatment failure. It was not developed to be a screening assay as not enough endemic controls or other endemic disease urines were used in the initial performance evaluation. The two ELISAs detect *Leishmania* antigens but with different sensitivities based on their cut- off for positivity (Table 3). Further refinement of the cut- off for positivity will be undertaken in future based on data obtained from an expanded panel of samples.

The *Leishmania* Antigen Detect™ ELISA and the *Leishmania* Antigen ELISA could serve as a standardized tool to measure the effectiveness of emerging treatment regimens in clinical trials and help make policies on implementation of new drug regimens in endemic regions [11]. Currently, splenic biopsy or PCR are used as a surrogate for parasite burdens in most trials involving new treatment regimens [26, 30]. Splenic biopsy is invasive, and not amenable for repeated sampling. PCR is expensive and hard to standardize among laboratories. As a direct detection test that reflects parasite burdens, the ELISAs could be the alternative with non- invasive sampling, high sensitivity and a quantitative read- out being distinct advantages.

Our study provides compelling data for further refinement of the *Leishmania* Antigen Detect™ ELISA for deployment in endemic regions for multiple VL management purposes. Further assessments with samples from endemic regions reflecting co- infections with TB or HIV and different treatment regimens are needed to qualify the *Leishmania* Antigen Detect™ ELISA for clinical use, including diagnosis and test of cure. Development of lateral flow assay formats of both ELISAs, which would be more suitable for community use should also be envisioned.

## Conclusions

The lack of standardized tools to monitor treatment hampers VL management in many ways. An effective tool that reflects parasite burden in VL: patients can help assess the suitability of the treatment regimen, foresee treatment failure and possibly predict post- treatment complications such as relapse. In this study, we have compared the suitability of two direct detection sandwich- ELISA based standardized tests, developed to detect *Leishmania*– specific antigens in the urine of patients. Both displayed high sensitivity and specificity on samples from the major endemic regions as well as, reflected parasite clearance in patients undergoing antimonial treatment in Ethiopia, a significant improvement over KATex, the only existing antigen detection test in the market. The ELISAs are user- friendly and quantitative and are suitable for deployment in routine care. They could also be adapted to a more cost- effective and point- of - care format such as lateral flow. Thus, we consider the tests a promising alternative to existing tests to monitor treatment and cure and worthy of further assessment and wide- spread deployment in endemic regions.

## Abbreviations

VL: Visceral Leishmaniasis
NEC: Non- endemic control
OD: Other diseases
*L. donovani*: *Leishmania donovani*
*L. infantum*: *Leishmania infantum*
HIV: Human immunodeficiency virus
ELISA: Enzyme-linked immunosorbent assay
SSG: Sodium Stibogluconate
MEG: Meglumine antimoniate
RDT: Rapid diagnostic test
WCL: Whole cell lysate
SLA: Soluble lysate antigen
HrP: Horse radish peroxidase
PKDL: Post- Kala azar dermal leishmaniasis
PCR: Polymerase chain reaction
UAU: Urinary antigen units
